# Vaccine against human Papilloma Virus

**Published:** 2014-09-30

**Authors:** Julio C Reina, Nubia Muñoz

**Affiliations:** 1 Profesor Titular y Emérito (J), Departamento de Pediatría, Universidad del Valle. Pediatra Investigador Centro Medico Imbanaco, Cali, Colombia; 2 Ex-jefe de Epidemiologia en la Agencia Internacional para la Investigación del Cáncer en Lyon, Francia y Profesora Emérita del Instituto de Cancerología de Colombia

At present two prophylactic human papilloma virus (HPV) vaccines are commercially available. The tetravalent vaccine comprised of four VPH types (6, 11, 16, and 18) distributed in the national program in Colombia and the bivalent vaccine comprised of VPH types 16 and 18, respectively. Types 16 and 18 are accountable for 70% of cervical cancer, most vulva, vagina, penis, anus and one third of oropharyngeal cancer. Types 6 and 11 are responsible for 90% of genital warts or condylomas and of recurrent laryngeal papillomatosis. Controlled clinical trials carried out in 30 countries with over 40,000 adolescents and young women have clearly showed that both vaccines are safe and prevent cervix cancer in situ with almost a 100% efficacy 1 and a decrease of genital warts and of high degree cervical intraepithelial neoplasia (CIN) lesions have been reported in national immunization programs.

The safety of both vaccines has periodically been assessed and they have been declared safe by the health authorities of several countries such as the Food and Drug Administration (FDA) and the Centers for Disease Control and Prevention (CDC) of the United States, the European Medicine Agency (EMEA) and the Global Advisory Committee on Vaccine Safety and the World's Health Organization (WHO).

Since the vaccination programs started, several safety and efficacy surveillance protocols for the vaccines have been implemented. Some are passive, such as the Vaccine Adverse Event Reporting System (VAERS) in the United States, which showed that the post-vaccination adverse event rates with the tetravalent vaccine were not higher than the historical rates of other vaccines 2. Others are the active surveillance such as the multicenter study in 7 health care organizations in the United States 3, on women between 9 and 26 years of age, who received 600,558 doses of tetravalent vaccine and the purpose of which was to monitor certain adverse events, such as: Guillain-Barre Syndrome, cerebrovascular accident (CVA), venous thromboembolism (VTE), appendicitis, convulsions, syncope, allergic reactions and anaphylaxis. No meaningful increase was found in the risk of predetermined objectives. In five cases of VTE, risk factors such as the use of oral contraceptives, blood clotting disorders, smoking habit, obesity and long hospitalization were involved. At Denmark and Sweden, Arnheim-Dahltröm *et al*. 4, studied a cohort consisting of 997,585 women between 10 and 17 years of age, from October 2006 to December 2010, which 296,826 received 696,420 doses of tetravalent vaccine. The studied adverse events were autoimmune, neurologic and VTE diseases during the following six months after vaccination. No evidences were found supporting the association of the exposition to the vaccine and the mentioned diseases. A multicentric study of cases and controls carried out between December 2007 and April 2011 in 113 specialized centers in France 5 investigated the possible association of the tetravalent vaccine and the risk of autoimmune disorders (AD): Idiopathic Thrombocytopenic Purpura (ITP), Central Demyelization and Multiple Sclerosis, Guillain-Barré Syndrome, Systemic Erythematous Lupus, Juvenile Rheumatoid Arthritis, type 1 Diabetes Mellitus, autoimmune Thyroiditis. No increase was observed in the autoimmune disorder risks studied. The Guillain-Barré and Thyroiditis cases found had not been exposed to the vaccine. 

The Global Advisory Committee on Vaccine Safety of the World's Health Organization (WHO) 6 in its report of March 2014 analyzed the evidence of the relationship between the Human Papillomavirus Vaccine with >175 million of doses distributed worldwide and autoimmune diseases, particularly Multiple Sclerosis, Aluminum as adjuvant, Vasculitis caused by vaccine DNA fragments and the Complex Regional Pain Syndrome described in Japan. The Committee ratified the strict vaccine safety control and based on a thorough examination of existing evidence, reaffirmed that the risk-benefit profile keeps favorable. Nevertheless there are some worries about the alarming statements based on anecdotic remarks and reports without any biological or epidemiological support in detriment of the community's healthcare.

The false alarms include aluminum and mercury. Adjuvant aluminum salts are important components of the vaccines, stimulating the immune system in order to respond in a more efficient manner to antigens. It is used as adjuvant (amorphous aluminium hydroxyphosphate sulphate) in the 0.225 mg tetravalent vaccine dose. Mitkus *et al*. 7, made an updated review of the Aluminum pharmacokinetic in breast-fed babies exposed through the diet and vaccines. It was concluded that the aluminum contained in the vaccine is less than in some diets and in some drugs. The broad immunization plan in Colombia (PAI) in the first year of life includes three doses of diphtheria, tetanus, acellular pertussis, hepatitis B, Polio I.M., Haemophilus influenza, pneumococcus and hepatitis B. The aluminum content of all those vaccines is of 2.22 mg.

Thimerosal, an organic composite of mercury is metabolized into ethyl-mercury and thiosalicylate, was used as preservation agent of vaccines to avoid its contamination with bacteria and fungus from 1930 to 1999. It was removed from vaccines because its possible relationship with neurodevelopment disorders in children. 

In Colombia, 10 years ago a group of about 200 adolescents, of both genders between 9 and 15 years, received three (3) doses of the tetravalent vaccine in a case research protocol and controls in Cali, Medellin and Bogotá. A strict follow-up has been made every six months by the researchers and no serious events have occurred regarding the vaccine, only minor events (pain, redness in the puncture site, fever, syncope) similar to those of other vaccines. The case of the children of Carmen de Bolivar in Colombia has been described by several authors in other countries as "Massive Psychogenic Event" 8, which has absolute no relationship with the vaccine but its high media dissemination resulted into disastrous consequences for the national vaccination program. 
